# Editorial: Nutrition regulation and stress in ruminant

**DOI:** 10.3389/fvets.2024.1456709

**Published:** 2024-07-19

**Authors:** Xianwen Dong, Juncai Chen, Zheng Zhou, Rui Hu

**Affiliations:** ^1^Chongqing Academy of Animal Science, Chongqing, China; ^2^College of Animal Science and Technology, Southwest University, Chongqing, China; ^3^Department of Animal Science, Michigan State University, East Lansing, MI, United States; ^4^Animal Nutrition Institute, Sichuan Agricultural University, Chengdu, China

**Keywords:** stress, ruminants, nutrition, immunity, rumen health

Stress was first introduced by Hans Selye in 1936 and has been defined as the body's systematic nonspecific adaptive response when stimulated by internal and external environmental and physiological factors. Ruminants inevitably face various stresses due to changes in physiological stages, environmental conditions and feeding management during the process of breeding production. Stress negatively impacts the nutrient digestibility and health status of ruminants, which ultimately leads to a decrease in production performance and economic efficiency, especially in young ruminants. The articles within this Research Topic detail the nutritional strategies to mitigate animal stress, and cover a wide-range of alternative feed ingredients or dietary supplements on rumen health, immunity and antioxidant function in ruminants at different physiological stages. It contributes to our current understanding of nutritional regulation to mitigate the negative effects of stress on animals.

There is a growing recognition within the livestock industry and society at large that animal stress is a significant issue that requires attention. With implications for animal health, wellbeing, and productivity, minimizing animal stress through improved animal management procedures and/or selective breeding is becoming a priority. With the development of biotechnology, more and more plant bioactive substances have been isolated and identified, some of which have been found to play a role in mitigating animal stress. On the other hand, Alkaline Mineral Complex (AMC) has been proven to have biological benefits and therapeutic effects on animals. The rumen serves as a vital digestive organ for ruminants and significantly influences their growth, performance, and overall health. Plant bioactive substances and AMC can induce changes in rumen function that impact the health and development of ruminants. To instigate readers' interest in the topic of nutritional regulation of animal stress, we present here the main findings reported by different authors in their nine manuscripts.

Plant bioactive substances are widely found in plants and are digested in the rumen used by ruminants. Starch in hydroponically barley seedlings (HBS) is metabolized into soluble polysaccharides during the growth period. Ma Y. et al. evaluated the effects of replacing different ratios of basal diets with HBS in lactating Hu ewes. The results indicated that HBS instead of 5%−15% of the basal diet were able to improve milk quality and alleviate oxidative stress in the body of ewes. Allium plants have been reported to have a positive effect on rumen fermentation and digestive capacity. Wang et al. found that the addition of 10 g/day *Allium mongolicum* Regel powder to the diet was beneficial for the stability of the rumen microbiota structure, which had a positive impact on the rumen health of Tibetan sheep lambs. Isatis Leaf (ISL) is a traditional Chinese herbal medicine that contains three main active compounds: indoles, quinazolones, and glucosinolates. The paper by Cao et al. studied the effect of ISL on the growth performance, gastrointestinal tissue morphology and microbiota of fattening sheep. In this study, the addition of 8% ISL to the diet increased rumen ammonia-nitrogen levels, regulated the gastrointestinal microbiota, promoted body fat metabolism, and enhanced immunity and resistance, thereby improving the health of fattening sheep. The improvement of rumen health by dietary composition more or less positively affects the health of sheep. However, supplementation of the plant bioactive substances in the form of feed ingredients requires the addition of larger doses.

Plant extracts have been used for thousands of years due to their high bioactive components, which have a positive effect on certain physiological activities of animals. It is also used as phytogenic feed additives to mitigate the negative effects of stress on ruminants. Feng et al. reported that the addition of 40 mg/kg body weight (BW) each day of *Dioscorea alata* L. anthocyanin (extracted from *Dioscorea alata* L.) to the diet improved antioxidant capacity, immune function and meat quality of Hainan black goats. Zhang et al. demonstrated that the addition of 30 mg/kg BW (sheep/day) ellagic acid (commercial product, purity ≥90%) to the diet of 5-month-old Kazakh sheep could improve dry matter intake and apparent digestibility of neutral detergent fiber and crude fat, increase the content of acetic acid and propionic acid in rumen fluid, regulate rumen microbiota, enhance antioxidant capacity and improve daily weight gain. Xu et al. examined the effects of tea polyphenols on growth performance, cytokine content, intestinal antioxidant status and intestinal barrier function of weaned lambs. The study demonstrated that 4–6 g/kg tea polyphenols (purity = 98.1%) could enhance the immunity and antioxidant capacity of lambs, improve the intestinal barrier function, reduce intestinal damage and protect intestinal health. Moreover, the dietary addition of tea polyphenols had anti-inflammatory and antioxidant effects similar to those of chlortetracycline. Ma X. et al. provide valuable scientific insights for the rational application of *Salvia sclarea* extract in lamb production by adding different levels of *Salvia sclarea* extract to the growth performance, serum immunity, and antioxidant indices of lambs. The addition of 0.12 ml/kg *Salvia sclarea* extract (essential oil, purity = 85%) to the diet improved the growth performance of lambs by increasing feed intake and nutrient digestibility. It also improved the health status of lambs by increasing their serum antioxidant capacity and immune function. Indeed, plant extracts offer the advantage of exerting their bioactive effects in small quantities, and their degradation in the rumen should be taken into account when used on adult ruminants.

Alkaline Mineral Complex is a complex mixture of alkaline ions that helps maintain the acid-base balance of rumen fluid in ruminants. Liu et al. investigated the effects of different concentrations of AMC on fermentation characteristics and bacterial composition *in vitro*. The addition of 2 ml/kg AMC to the substrate stabilized the rumen environment by increasing the fatty acids concentration in fermentation fluid, without altering the rumen microbiota. Ulteriorly, Guo et al. demonstrated that supplementation with AMC water could improve immune function and antioxidant capacity of calves, and reduce diarrhea.

This Research Topic of articles describe a variety of nutritional strategies to alleviate animal stress, with plant bioactive substances accounting for the majority of the research. In most of these papers, rumen function and health were positively affected by the dietary treatments, resulting in an improvement of ruminant health. These findings provide theoretical support for the development of nutritional regulation of stress resistance in ruminants.

## Author contributions

XD: Supervision, Writing – original draft, Writing – review & editing. JC: Validation, Writing – original draft, Writing – review & editing. ZZ: Supervision, Validation, Writing – review & editing. RH: Supervision, Validation, Writing – review & editing.

